# Estimated cardiorespiratory fitness in childhood and cardiometabolic health in adulthood: 1970 British Cohort Study

**DOI:** 10.1111/sms.13637

**Published:** 2020-02-26

**Authors:** Mark Hamer, Gary O’Donovan, G. David Batty, Emmanuel Stamatakis

**Affiliations:** ^1^ Division of Surgery & Interventional Science Faculty of Medical Sciences Institute Sport Exercise Health University College London London UK; ^2^ Facultad de Medicina Universidad de los Andes Bogotá Colombia; ^3^ Department of Epidemiology & Public Health University College London London UK; ^4^ School of Biological & Population Health Sciences Oregon State University Corvallis OR USA; ^5^ Charles Perkins Centre The University of Sydney Sydney NSW Australia; ^6^ School of Public Health Prevention Research Collaboration The University of Sydney Sydney NSW Australia

**Keywords:** cohort, fitness, metabolic, population

## Abstract

**Background:**

Associations of cardiorespiratory fitness in childhood and adulthood with adult cardiometabolic risk factors are poorly understood, not least because of the paucity of studies.

**Objectives:**

We investigated associations between nonexercise testing cardiorespiratory fitness (NETCRF) in childhood/adulthood and cardiometabolic risk factors in adulthood.

**Methods:**

Based on an established algorithm comprising gender, age, body mass index, resting heart rate, and self‐reported physical activity at age 10, we computed NETCRF. Risk factors were assessed at age 46 in 5009 participants when NETCRF was again calculated. Linear regression was used to summarize associations between NETCRF in childhood and risk factors in adulthood and, additionally, the relationship between NETCRF in adulthood and risk factors in adulthood after adjusting for childhood NETCRF.

**Results:**

Inconsistent associations were observed between childhood NETCRF and adult risk factors. NETCRF in adulthood was associated with blood pressure [−5.8 (−6.7, −4.9)], glycated hemoglobin [−3.41 (−4.06, −2.76)], total cholesterol [−0.16 (−0.24, −0.08)], HDL cholesterol [0.19 (0.16, 0.22)], triglycerides [−0.68 (−0.85, −0.50)], and C‐reactive protein [−0.29 (−0.35, −0.22)] in adult males. Similar associations were observed in adult females. Compared to those with low estimated fitness in both childhood and adulthood, participants with low fitness in childhood and high fitness in adulthood had a lower risk of two or more cardiometabolic risk factors (odds ratio: 0.25; 95% confidence interval: 0.19, 0.31).

**Conclusion:**

Associations between estimated fitness and risk factors are stronger in adulthood than from childhood to adulthood. Adults with previously sedentary childhoods may still gain benefits from improving their fitness.

## 1 INTRODUCTION

Cardiorespiratory fitness quantifies the functional capacity of an individual and reflects the ability of the lungs and cardiovascular system to transport oxygen and the ability of the tissues and organs to extract and use oxygen during sustained physical activity. The associations between cardiorespiratory fitness and health in adults are well understood.^1^ Indeed, the consensus is that there are inverse associations between cardiorespiratory fitness and morbidity and mortality.^1^ The associations between cardiorespiratory fitness and health in children are less well understood.^2^, ^3^ For example, more research is required to better understand the associations between cardiorespiratory fitness in childhood and cardiometabolic risk factors in adulthood,^2^, ^3^ and if fitness accrued through adulthood can counteract poor fitness in childhood.

Cardiorespiratory fitness can be directly measured in clinical settings or estimated from field tests and nonexercise equations.^1^, ^4^, ^5^ One of the main objectives of this study is to investigate associations between nonexercise testing cardiorespiratory fitness (NETCRF) in childhood and cardiometabolic risk factors in adulthood. The other main objective of this study is to investigate associations between NETCRF in adulthood and cardiometabolic risk factors in adulthood after adjustment for NETCRF in childhood.

## 2 METHODS

### 2.1 Participants

The 1970 British Birth Cohort Study consists of people born in England, Scotland, and Wales during a single week in 1970.^6^, ^7^ The present analysis includes data from the age 10 (1980‐1981) and age 46 (2016‐2018) surveys.^8^, ^9^ At the age 10 survey, health visitors went to the homes of cohort members and conducted interviews with parents.^8^ Parents and cohort members were also asked to complete questionnaires.^8^ A simple medical assessment also took place in the cohort member's home or school.^8^ At the age 46 survey, health visitors or nurses went to the homes of cohort members and conducted interviews and computer‐assisted interviews with cohort members.^9^ A medical assessment also took place in the cohort member's home and the nurse obtained a non‐fasting blood sample.^9^ Parents (age 10 survey) and cohort members (age 46 survey) provided informed consent and the age 46 survey was approved by the NRES Committee South East Coast ‐ Brighton & Sussex (Ref 15/LO/1446).

### 2.2 Variables at age 10

At the age 10 survey, the cohort member's parent was asked how often their child played sport in his or her spare time.^8^ The available responses were as follows: never or hardly ever, sometimes, and often.^8^ The health visitor measured height and weight and body mass index (BMI) was calculated.^8^ The child was settled for two minutes and the health visitor measured resting heart rate (RHR) at the wrist for one minute while the child was sitting using the palpation method.^8^ Parents provided information about their occupation, which was categorized according to the 1970 and 1980 Office of Population Censuses and Surveys Classification of Occupations: managerial or professional; intermediate (skilled & non‐skilled); and routine or manual.^8^

### 2.3 Variables at age 46

A modified version of the EPIC Physical Activity Questionnaire was used to assess leisure‐time physical activity.^9^, ^10^ A nurse measured height and weight and BMI was calculated.^9^ The nurse also measured blood pressure and RHR using an automated device (HEM 907, Omron Healthcare, Milton Keynes, UK): The nurse fitted an appropriately sized cuff to the respondent's right arm; the respondent rested for five minutes; and three measurements of blood pressure and RHR were taken at one‐minute intervals.^9^ We calculated the average blood pressure from second and third readings after discarding the first. The nurse also collected a non‐fasting blood sample.^9^ Standard enzymatic methods were used to assess total cholesterol, high‐density lipoprotein (HDL) cholesterol, and triglycerides concentrations.^9^ Ion exchange high‐performance lipid chromatography was used to assess glycated hemoglobin HbA1C concentrations, and immunoturbidimetry was used to assess high‐sensitivity C‐reactive protein (hsCRP) concentrations.^9^ The coefficients of variation of the assays were as follows: 0.7%‐2.6% for total cholesterol and for HDL cholesterol; 0.8%‐2.4% for triglycerides; 0.6%‐3.3% for HbA1C; and 2.3%‐10.5% for hsCRP.^9^ Alcohol consumption was evaluated using the Alcohol Use Disorders Identification Test—Primary Care Version (AUDIT‐PC) that consists of 5 questions covering alcohol consumption, problems, and dependency. Responses to each question are scored from 0 to 4 giving a maximum score of 20. Scores of 5 or more are associated with increasing or higher risk drinking. Cohort members also provided data on cohabiting status, and highest educational attainment.

### 2.4 Calculation of NETCRF

Previous work suggested that cardiorespiratory fitness may be accurately estimated in adults from a nonexercise test model including gender, age, body mass index, resting heart rate, and self‐reported physical activity; the algorithm has demonstrated strong concurrent validity (*r*'s 0.76‐0.81) against exercise testing‐estimated fitness.^11^ Nonexercise testing cardiorespiratory fitness estimates were converted into maximal aerobic capacity metabolic equivalent (MET) values such that one MET corresponds to an oxygen consumption of 3.5 mL/kg/min (based on a 70 kg man aged 40 years). The algorithm was calculated as follows 12: NETCRF = [sex coefficient × 2.78 – (age × 0.11) – (BMI × 0.17) – (RHR × 0.05) + (physical activity level coefficient) + 21.41]. The sex coefficient was 1 for men and 0 for women. The physical activity coefficients at age 10 were based on leisure‐time sports participation: 0.0 for never or hardly ever; 0.29 for sometimes; and, 1.21 for often. The physical activity coefficients at age 46 were based on adherence to contemporaneous guidelines 13, 14 assessed using the EPIC Physical Activity Questionnaire: 0.0 for inactive during leisure‐time; 0.29 for active, but not meeting the guidelines; and, 1.21 for meeting the guidelines of at least 150 minutes per week of moderate‐intensity or 75 minutes per week of vigorous‐intensity physical activity.

### 2.5 Statistical analysis

Linear regression was used in the main analysis, with separate models for males and females. First, linear regression was used to investigate associations between NETCRF in childhood and risk factors in adulthood, adjusting for medication 15: A constant of +18% was added to the original value of triglycerides and −5% to the original value of HDL cholesterol in those on lipid‐lowering drugs; a constant of +10 mm Hg was added to the original systolic and diastolic blood pressure values in those treated for high blood pressure; and, a constant of +11 mmol/mol was added to the original value of HbA1C in those taking oral medication for type 2 diabetes. Second, linear regression was used to investigate associations between NETCRF in adulthood and risk factors in adulthood, adjusting for NETCRF in childhood. Logistic regression was used in the secondary analysis in order to investigate changes. Cohort members were split into low‐ or high‐fitness groups using the sex‐specific median split for NETCRF in childhood and adulthood, creating four categories of childhood and adulthood estimated fitness: low and low; low and high; high and low; and, high and high. These change categories were then regressed onto a binary metabolic risk outcome defined as the presence of two or more of the following risk factors^16^: high blood pressure (≥130/80 mm Hg); low HDL‐cholesterol (<1.03 mmol/L in men and <1.30 mmol/L in women); high triglycerides (≥1.7 mmol/L); impaired glycaemic control (HbA1C > 6.0% (42.1 mmol/mol)); or, systematic inflammation (hsCRP ≥ 3 mg/L). Linear and logistic regression models were adjusted for parental occupational social class in childhood, highest educational attainment, cohabiting status, problematic alcohol, and smoking in adulthood. All analyses were conducted in SPSS version 22 (IBM Inc).

## 3 RESULTS

### 3.1 Participants’ characteristics

At the age 10 survey, 11 938 cohort members provided the information required to calculate NETCRF, including BMI, RHR, and physical activity. Of these cohort members, 5009 attended the age 46 survey and provided blood samples. Missing cohort members were more likely to be male (54.7% vs 48.4%, *P* = .001), were more likely to have higher BMI in childhood (16.9 vs 16.8 kg/m^2^, *P* = .02) and were more likely to have fathers in routine and manual occupations (18.7% vs. 13.6%, *P* = .001). Table 1 shows participants’ characteristics.

**Table 1:** Characteristics of the sample

	Male	Female
Resting HR age 10 (bpm)	79.9 ± 10.8	82.8 ± 11.4
Body mass index age 10 (kg/m^2^)	16.7 ± 1.9	17.0 ± 2.3
Sports participation age 10 (%)		
Never	4.8	10.9
Occasional	28.3	47.8
Frequent	66.8	41.3
NETCRF age 10 (METS)	17.1 ± 0.8	13.9 ± 0.9
Resting HR age 46 (bpm)	67.7 ± 11.3	69.1 ± 10.2
Body mass index age 46 (kg/m^2^)	28.6 ± 4.6	28.2 ± 6.1
Physical activity^a^ age 46 (%)		
None	37.6	35.7
Active below guideline	10.8	12.8
Meeting guidelines	51.6	51.5
NETCRF age 46 (METS)	11.5 ± 1.3	8.8 ± 1.5
Smoking in adulthood (%)		
Never	46.8	48.5
Ex‐smoker	31.4	31.7
Current	21.9	19.8
High‐risk alcohol (%)	34.2	19.1
Cohabiting with partner (%)	77.0	74.5
Degree educated (%)	26.9	28.1
Medication^b^ (%)	11.3	7.8
Systolic BP (age 46) (mmHg)	128.8 ± 13.5	119.6 ± 15.1
Total cholesterol (mmol/L)	5.5 ± 1.1	5.3 ± 0.9
HDL cholesterol (mmol/L)	1.4 ± 0.4	1.7 ± 0.4
Triglycerides (mmol/L)	2.3 ± 1.7	1.5 ± 0.9
Glycated hemoglobin (mmol/mol)	37.6 ± 8.6	36.2 ± 7.2
C‐reactive protein (mg/L)^c^	1.0 (2.0)	1.0 (2.3)

Note

Data are presented as mean (SD) unless otherwise denoted.

a Meeting physical activity guidelines include ≥ 150 min/wk moderate activity or ≥ 75 min/wk vigorous.

b Includes anti‐hypertensives, lipid‐lowering, and blood glucose control.

c Median and interquartile range.

### 3.2 NETCRF in childhood and risk factors in adulthood

Table 2 shows the associations between NETCRF in childhood and cardiometabolic risk factors in adulthood. NETCRF in childhood was associated with HDL cholesterol in adult males and blood pressure, glycated hemoglobin, HDL cholesterol, triglycerides, and C‐reactive protein in adult females.

**Table 2:** Association between NETCRF age 10 and cardiometabolic risk factors in adulthood

	Male B (95% CI)	Female B (95% CI)
Systolic blood pressure (n = 5009)	−0.2 (−1.5, 1.0)	−3.0 (−4.3, −1.7)
HbA1C (n = 3989)	−0.97 (−1.97, 0.03)	−0.80 (−1.55, −0.06)
Total cholesterol (n = 4025)	0.05 (−0.01, 0.11)	−0.01 (−0.06, 0.04)
HDL cholesterol (n = 4025)	0.04 (0.002, 0.08)	0.06 (0.02, 0.11)
Triglycerides (n = 2234)	−0.05 (−0.29, 0.20)	−0.21 (−0.32, −0.10)
Log C‐reactive protein (n = 2207)	−0.05 (−0.13, 0.04)	−0.12 (−0.20, −0.03)

Note

Data presented per SD unit increase in NETCRF. Coefficients are adjusted for smoking in adulthood, problematic alcohol consumption, cohabiting status, cohort member highest educational attainment, and father's social occupational status.

### 3.3 NETCRF in adulthood and risk factors in adulthood

Table 3 shows the associations between NETCRF in adulthood and cardiometabolic risk factors in adulthood. NETCRF in adulthood was associated with blood pressure, glycated hemoglobin, total cholesterol, HDL cholesterol, triglycerides, and C‐reactive protein in adult males and adult females after adjustment for NETCRF in childhood, and other covariates.

**Table 3:** Association between NETCRF age 46 and cardiometabolic risk factors

	Male B (95% CI)	Female B (95% CI)
Systolic blood pressure (n = 5009)	−5.8 (−6.7, −4.9)	−6.0 (−6.9, −5.1)
HbA1C (n = 3989)	−3.41 (−4.06, −2.76)	−2.44 (−2.91, −1.96)
Total cholesterol (n = 4025)	−0.16 (−0.24, −0.08)	−0.17 (−0.23, −0.11)
HDL cholesterol (n = 4025)	0.19 (0.16, 0.22)	0.22 (0.19, 0.25)
Triglycerides (n = 2234)	−0.68 (−0.85, −0.50)	−0.31 (−0.38, −0.24)
Log C‐reactive protein (n = 2207)	−0.29 (−0.35, −0.22)	−0.42 (−0.47, −0.37)

Note

Data presented per SD unit increase in NETCRF. Coefficients are adjusted for NETCRF age 10, smoking in adulthood, problematic alcohol consumption, cohabiting status, cohort member highest educational attainment and father's social occupational status

### 3.4 Changes in NETCRF and cardiometabolic health

We estimated changes in estimated fitness from childhood to adulthood and the data suggested gaining or maintaining a high level of fitness was advantageous (Table 4). For example, compared with those with low estimated fitness in childhood and low estimated fitness in adulthood (reference group), the odds ratio for the presence of two or more cardiometabolic risk factors was 0.25 (95% confidence interval: 0.19, 0.31) in those with low estimated fitness in childhood and high estimated fitness in adulthood.

**Table 4:** Association between change in NETCRF from childhood to adulthood and metabolic health

NETCRF age 10	NETCRF age 46	Cases/total N	Presence of > 1 metabolic risk factor^a^ Odds ratio^b^ (95% CI)
Low	Low	296/667	1.0 (Ref)
Low	High	73/510	0.25 (0.19, 0.31)
High	Low	217/588	0.84 (0.66, 1.11)
High	High	81/624	0.27 (0.22, 0.34)

a Metabolic risk factors: high BP (BP ≥ 130/80 mmHg), impaired glycaemic control (HbA1c > 6.0% [42.1 mmol/mol]), systemic inflammation (CRP ≥ 3mg/L), low HDL cholesterol (<1.03 mmol/L in men and <1.30 mmol/L in women), and high triglycerides (≥1.7 mmol/L).

b Model adjusted for sex, smoking, problematic alcohol consumption, cohabiting status, cohort member highest educational attainment, fathers social occupational status.

### 3.5 Sensitivity analyses

While there will of course be individual variation in response to medications, the addition of constant values are known average changes in population‐based studies. We repeated analyses after removal of participants on medication although associations between NETCRF and risk markers were not appreciably changed (see Table S1). Just over a third of the sample were obese (BMI ≥ 30 kg/m^2^); in analyses stratified by obesity status associations between NETCRF and risk factors were observed in both obese and non‐obese cohort members (Table S2).

## 4 DISCUSSION

One of the main objectives of this study was to investigate associations between NETCRF in childhood and cardiometabolic risk factors in adulthood. We found that NETCRF in childhood was associated with some cardiometabolic risk factors in middle‐aged adults, particularly in women. The other main objective of this study was to investigate associations between NETCRF in adulthood and cardiometabolic risk factors in adulthood after adjustment for NETCRF in childhood. We found that NETCRF at age 46 was associated with blood pressure and blood‐borne cardiometabolic risk factors at age 46 after adjustment for NETCRF at age 10. Studies of children of different ethnic groups suggest that it is plausible that genes explain at least some of the association between fitness and health in childhood.^17^ Studies of adults suggest that it is plausible that habitual physical activity explains at least some of the association between fitness and health in adulthood.^1^

The present study is one of the largest studies with data across the life course to examine the associations between estimated cardiorespiratory fitness in childhood and cardiometabolic risk factors in adulthood.^2^ In other recent work, cardiorespiratory fitness was estimated in a maximal cycling test in some 1.5 million 18‐year‐olds and the authors found that low fitness was associated with increased risk of type 2 diabetes during up to 46 years of follow‐up.^18^ Associations between cardiorespiratory fitness in childhood and indices of glucose metabolism in young adulthood were investigated in 317 participants in the European Youth Heart Study.^19^ Cardiorespiratory fitness was estimated via a maximal cycling test at age 15 years, and fasting blood samples were drawn at age 15 and at age 21 or age 27.^19^ The authors found that higher cardiorespiratory fitness was associated with favorable homeostasis model assessment of insulin resistance and ß‐cell function values after 6‐12 years of follow‐up.^19^ Associations between cardiorespiratory fitness in childhood and cardiometabolic risk factors in adulthood were also considered in a review of studies that included 38 articles, assessing 44,169 children and adolescents followed up for a median of 6 years. 2; There was considerable heterogeneity in methodology, measurement of CRF, and outcomes, which hampered the quality of the evidence. There was evidence for associations of higher childhood CRF with lower BMI, waist circumference, body fatness, and lower prevalence of metabolic syndrome in later life, but only in around half the studies. No consistent associations between childhood CRF and future waist‐to‐hip ratio, blood pressure, lipid profile, and glucose homeostasis were observed.^2^ Thus, the mixed findings are largely consistent with the present results in relation to childhood fitness and adult risk markers.

In the present study, there were limited associations between estimated fitness in childhood and risk factors in adulthood, yet more consistent associations between estimated fitness in adulthood and risk factors in adulthood after adjustment for fitness in childhood. Gaining or maintaining a high level of fitness was also advantageous. Although physical activity and cardiorespiratory fitness are different concepts, they are likely to be interrelated as participation in regular moderate‐vigorous physical activity directly improves cardiorespiratory fitness.^20^ Thus, the results of the present study may have implications for practitioners and policy makers. The importance of assessing cardiorespiratory fitness in adults is well recognized^1^; And, the present study suggests that associations between estimated cardiorespiratory fitness and cardiometabolic risk factors are stronger in adulthood than from childhood to adulthood. More research is required to determine whether physical activity or cardiorespiratory fitness is the more important risk factor in children^21^; nonetheless, it could be argued that children should be made aware of their physical activity habits so that they may maintain or develop the moderate‐ and vigorous‐intensity physical activities that are associated with fitness and health in adults.^22^ It is important to emphasize that gains in cardiorespiratory fitness can be quickly lost if exercise is not maintained.^23^ Some adults may be resistant to the HDL‐raising effects of exercise training^24^; however, physical activity is associated with reduced risk of all‐cause mortality in adults with low HDL‐cholesterol concentrations.^25^

This study has some limitations. Nonexercise equations are a useful research tool and most men and women are correctly classified into low‐ or high‐fitness groups^26^; however, nonexercise equations should not be viewed as a replacement for the direct measurement of cardiorespiratory fitness in patients in clinical settings.^1^ The nonexercise equation used in the present study has been well validated in adults,^11^ compared to other approaches such as self‐reported functional capacity/ fitness.^27^, ^28^ There are a range of NETCRF equations in use and the most basic equations include age, sex, and anthropometric variables, although more advanced equations also include measures of body composition and physical activity.^29^ When physical activity was included the equation typically provided estimated values that were better correlated to directly measured fitness.^29^ Nevertheless, NETCRF equations do not account for genetic influences on fitness and some variables are self‐reported, including physical activity, and are subject to biases. Obtaining objective assessments of activity are unlikely to be feasible and practical in most clinical settings. Similar equations have been validated in youths (there were no statistically significant differences in cardiorespiratory fitness when estimated from nonexercise equations and when estimated from a submaximal step test in 30 males and 30 females aged 17‐22 years).^5^ Cardiorespiratory fitness may moderate the association between overall physical activity and cardiometabolic risk factors in children,^30^ but physical activity was crudely assessed in children in the present study. The vast majority of participants in the 1970 British Birth Cohort Study are white,^6^ and the present study may not be generalizable to other groups. Risk markers were not measured at baseline, prohibiting a true longitudinal analysis, although biomarkers would have been within the normal healthy range in the majority of the sample at the age 10 survey.

## 5 PERSPECTIVE

There were few associations between NETCRF in childhood and risk factors in adulthood in the present study yet more consistent associations emerged between adulthood NETCRF and risk factors after adjustment for fitness in childhood. This is one of the longer and larger studies of its kind and the results suggest that associations between NETCRF and cardiometabolic risk factors are stronger in adulthood than from childhood to adulthood. Adults with previously sedentary lifestyles and low fitness may still gain health benefits from improving their fitness in mid‐life.

## CONFLICT OF INTEREST

None declared.
