# Supplementary material for: Estimated cardiorespiratory fitness in childhood and cardiometabolic health in adulthood: 1970 British Cohort Study
Source: Scand J Med Sci Sports. 2020 Feb 26;30(5):932–8. doi: 10.1111/sms.13637 (PMC7187251; doi:10.1111/sms.13637)
Supplement: Supplementary file 1 [file SMS-30-932-s001.docx]

**Supplementary analyses**

Table S1. Association between NETCRF age 46 and cardiometabolic risk factors after removing participants taking medication.

|  | Male  B (95% CI) | Female  B (95% CI) |
| --- | --- | --- |
| Systolic blood pressure | -4.9 (-5.8, -3.9) | -4.9 (-5.8, -4.0) |
| HbA1C | -1.56 (-1.96, -1.17) | -1.46 (-1.77, -1.16) |
| Total cholesterol | -0.23 (-0.32, -0.14) | -0.19 (-0.25, -0.13) |
| HDL cholesterol | 0.20 (0.16, 0.23) | 0.22 (0.19, 0.25) |
| Triglycerides | -0.63 (-0.80, -0.47) | -0.30 (-0.37, -0.22) |
| Log C-reactive protein | -0.29 (-0.36, -0.22) | -0.42 (-0.42, -0.37) |

Data presented per SD unit increase in NETCRF.

Coefficients are adjusted for NETCRF age 10, smoking in adulthood, problematic alcohol consumption, cohabiting status, cohort member highest educational attainment and father’s social occupational status

Table S2. Association between NETCRF age 46 and cardiometabolic risk factors stratified by obesity.

|  | Non-obese (BMI<30 kg/m^2^)  B (95% CI) | Obese (BMI≥30 kg/m^2^)  B (95% CI) |
| --- | --- | --- |
| Systolic blood pressure | -4.6 (-5.6, -3.6) | -4.5 (-5.9, -3.1) |
| HbA1C | -1.32 (-1.82, -0.82) | -4.02 (-5.12, -2.90) |
| Total cholesterol | -0.21 (-0.29, -0.14) | 0.03 (-0.08, 0.14) |
| HDL cholesterol | 0.21 (0.18, 0.24) | 0.05 (0.02, 0.09) |
| Triglycerides | -0.59 (-0.73, -0.44) | -0.15 (-0.32, 0.03) |
| Log C-reactive protein | -0.27 (-0.32, -0.21) | -0.28 (-0.36, -0.19) |

Data presented per SD unit increase in NETCRF.

Coefficients are adjusted for sex, NETCRF age 10, smoking in adulthood, problematic alcohol consumption, cohabiting status, cohort member highest educational attainment and father’s social occupational status
